# Directing cell delivery to murine atherosclerotic aortic lesions via targeting inflamed circulatory interface using nanocarriers

**DOI:** 10.3389/fcvm.2025.1517320

**Published:** 2025-06-24

**Authors:** Carlos Theodore Huerta, Leiming Zhang, Yulexi Y. Ortiz, Yan Li, Elnaz Zeynaloo, Emre Dikici, Teruna J. Siahaan, Sapna K. Deo, Sylvia Daunert, Zhao-Jun Liu, Omaida C. Velazquez

**Affiliations:** ^1^ DeWitt Daughtry Family Department of Surgery, University of Miami Miller School of Medicine, Miami, FL, United States; ^2^ Department of Biochemistry and Molecular Biology, School of Medicine, University of Miami, Miami, FL, United States; ^3^ Dr. JT Macdonald Biomedical Nanotechnology Institute, University of Miami, Coral Gables, FL, United States; ^4^ Department of Pharmaceutical Chemistry, University of Kansas, Lawrence, KS, United States; ^5^ Vascular Biology Institute, University of Miami Miller School of Medicine, Miami, FL, United States

**Keywords:** stem cell therapy, cell-based therapy, atherosclerosis, targeted cell delivery, ICAM-1/LFA-1, nanocarriers

## Abstract

Stem cell therapy holds significant potential for many inflammatory diseases and regenerative medicine applications. However, delivery of therapeutic cells to specific disease sites after systemic administration without indiscriminate trafficking to other non-target tissues is a major limitation of current cell therapies. Here, we describe a novel nanocarrier-directed targeted cell delivery system that enables cell surface coating with dendrimer nanocarriers containing adhesion moieties to serve as a global positioning system “GPS” to guide circulating cells to targeted lesions and mediate the anchoring of cells at the inflammation site. By exploiting cell surface ligands/receptors selectively and/or molecular moieties that are highly expressed on activated endothelium in pathologic disease states, nanocarrier-coated cells containing the counterpart binding receptors/ligands can be enabled to specifically traffic to and dock at vasculature within target lesions. We demonstrate the efficacy of the I-domain fragment of LFA-1 (*id*LFA-1) complexed to modified nanocarriers to facilitate the homing of mesenchymal stem cells (MSCs) to inflamed luminal endothelial cells on which ICAM-1 is highly expressed in a murine model of aortic atherosclerosis. Our method can overcome challenges imposed by the high velocity and dynamic circulatory flow of the aorta to successfully deliver MSCs to atherosclerotic regions and allow for the docking of the potentially therapeutic and immunomodulating cells. This targeted cell delivery platform can be tailored for selective systemic delivery of various types of therapeutic cells to different disease areas.

## 1 Introduction

The effectiveness of stem cell therapy is contingent upon effective cellular engraftment and homing to diseased tissues to reestablish function and homeostasis. Administration of stem cells is most commonly accomplished via local injection to the site of disease or intravascular delivery, including intraarterial injection that quickly delivers therapeutic cells to the tissue/organs fed by a given artery, and intravenous routes, by which therapeutic cells are infused into the bloodstream (systemic delivery) (1, 2). However, these delivery routes are associated with substantial limitations. The most commonly employed administration route is direct injection, but cell-based therapy is significantly limited by cell viability and retention even when locally dispensed to sites of disease (1, 3). Furthermore, cells delivered via local injection may not function appropriately given environmental factors at the tissue level including hypoxia, poor blood flow, hyperglycemia, and widespread local inflammation associated with disease (4–7). Limited space and even physical pressure at sites of inoculation may further change cellular characteristics or hamper cell viability (5, 8, 9). Unfortunately, many disease sites are not amenable to access by local inoculation given their intracavitary location (brain, chest, abdomen, aorta, etc.), which would necessitate more invasive injection procedures. Systemic administration via intravenous dissemination can partially overcome this limitation in delivery to sites that are traditionally hard to access; however, it can lead to indiscriminate trafficking of cells and often results in a low number of cells delivered to diseased tissue (1, 10). Consequently, there is a critical, unmet need for novel methods to enhance the delivery of a sufficient number of therapeutic cells to diseased tissue sites.

At the circulatory system interface, endothelial cells (ECs) form a selectively permeable luminal barrier between blood and the neighboring tissue in homeostatic conditions. After physiological insult from tissue injury, malignancy, and/or inflammation, the release of numerous chemokines and cytokines such as TGF-β and SDF1-α activates neighboring ECs (11, 12). Resultant stimulation by these soluble factors can cause a variety of cell adhesion molecules (CAMs) such as integrins and selectins to be upregulated within the endothelium in the diseased tissue, thereby making the endothelial lining “sticky” and increasing its ability to tether circulating cells responding to tissue repair, inflammation, and immunomodulation signals (12).

Intercellular adhesion molecule (ICAM)-1 is a transmembrane cell surface molecule expressed by multiple cell types such as ECs. ICAM-1 plays essential roles in both innate and adaptive immune responses, as well as the recruitment of circulating leukocytes for trans-endothelial migration and inflammation. While ICAM-1 is constitutively expressed at a low basal level on ECs, its levels are augmented by inflammatory cytokines (TNF-α, IL-1, and IFN-γ) in diseased states such as atherosclerosis. Atherosclerosis-associated cardiovascular diseases (CVDs) are the leading cause of morbidity and mortality globally and claim the lives of over 17 million people annually (13–15). Previously thought to be a bland lipid storage disease, atherosclerosis is now more appropriately characterized by its chronic inflammatory nature (16). Moreover, mesenchymal stem cells (MSCs) exhibit noteworthy anti-inflammatory properties (17, 18), providing new options for the treatment of inflammatory diseases. Hence, targeted delivery of exogenous therapeutic MSCs to atherosclerotic lesions can be a potential therapeutic option.

Crucial interactions between immune cells and orchestrated CAM levels on arterial ECs such as ICAM-1 are thought to be an early event in the natural history of atherosclerotic lesions, as well as in the migration of such cells to subendothelial segments and development into foam cells (19, 20). Several ligands can bind to ICAM-1 of which the predominant counter-receptor mediating leukocyte interaction and recruitment is leukocyte function-associated antigen (LFA)-1. Specifically, the α-subunit of LFA-1 includes an amino terminus, I-domain, which is responsible for LFA-1 binding to ICAM-1 (21–24). ICAM-1 signaling transduction further contributes to the proinflammatory milieu and activation of ECs in blood vessel walls which propagate atherosclerotic plaque development (19, 25). Preclinical evidence has shown significant upregulation of ICAM-1 at atherosclerotic-prone sites in murine models of cardiovascular disease that are homozygous apolipoprotein E-deficient (ApoE^−/−^), particularly within the aorta (25, 26). Conversely, deficiency of ICAM-1 is protective against atherosclerosis and associated with substantially diminished atheromatous plaque burden *in vivo* (27–29). Consequently, the interaction between the I-domain of LFA-1 (*id*LFA-1) and ICAM-1 serves as an attractive therapeutic target for atherosclerotic disease of the circulatory system.

Herein, we describe a novel application of nanocarriers with programmed molecular moieties based on the interaction of *id*LFA-1/ICAM-1 to control the localization and delivery of therapeutic stem cells to target disease regions. Specifically, fifth-generation poly(amidoamine) (PAMAM) dendrimer nanoparticles were complexed with an adhesive moiety, *id*LFA-1, to form nanocarriers. In turn, these were utilized to install *id*LFA-1 on the surface of MSCs, which normally do not express LFA-1. Given our data and the previously mentioned expression pattern of ICAM-1 in luminal ECs at areas of atherosclerosis, *id*LFA-1 was selected as a targeting signal to enhance the docking and capture of the systemically delivered cells on the endothelial lumen at regions of vascular vessels expressing elevated levels of ICAM-1. Dendrimer nanoparticles complexed with the adhesion molecule of interest were physically incorporated on the cell surface by complexation via ionic interactions between positively charged dendrimer nanoparticles and negatively charged cell membranes of MSCs. Furthermore, PAMAM dendrimers were optimized by acetylation to minimize intracellular internalization and toxicity and maximize cell surface coating as previously described (5). MSCs coated with *id*LFA-1 modified nanocarriers can be then recruited to target tissue sites expressing the cognate binding ligand (in this case ICAM-1 on endothelial lumen in atherosclerotic lesions). In our work, we demonstrated the ability of both the target protein moiety and modified nanocarriers to bind ECs *in vitro* followed by the efficient, selective delivery of nanocarrier-coated MSCs *in vivo* to atherosclerotic lesions in the aorta, thereby reducing indiscriminate migration to other organs in the ApoE^−/−^ mouse model of atherosclerosis. Hence, this nanocarrier technology provides a highly targeted biocompatible strategy to augment tissue homing of MSCs that can be applied to numerous other therapies ranging from cardiovascular and autoimmune diseases to regenerative medicine applications.

## 2 Methods

### 2.1 Reagents

Magnesium chloride, magnesium sulfate, sodium phosphate dibasic, potassium phosphate monobasic, sodium chloride, potassium chloride, ethylene diamine tetraacetic acid (EDTA), Tris−HCl, Tween 20, benzamidine hydrochloride, glycerol, ampicillin, and sodium bicarbonate were purchased from Sigma-Aldrich (St. Louis, MO, USA). VivoTag XL 680 NHS ester was purchased from PerkinElmer. Protein-free blocking buffer, dithiothreitol (DTT), isopropyl-β-D-thiogalactopyranoside (IPTG), ProBlock Gold protease inhibitor, and guanidine hydrochloride were obtained from G-Biosciences (St. Louis, MO, USA). Pierce 1-Step Ultra TMB-ELISA, Pierce BCA Protein Assay Kit, *E. coli* BL21 cells, LB broth, and the Opti-MEM media were acquired from Thermo Fisher Scientific (Waltham, MA, USA). Primary antibody mouse IgG1 anti-human CD11a was purchased from BioLegend (San Diego, CA, USA). The secondary antibody was horseradish peroxidase (HRP)-conjugated anti-mouse IgG antibody and was procured from SouthernBiotech (Birmingham, AL, USA). KPL TMB Stop Solution was purchased from SeraCare (Milford, MA, USA). A 20% acetylated, generation 5 poly(amidoamine) (PAMAM) dendrimer was purchased from 21st Century Biochemicals (Marlborough, MA, USA).

### 2.2 Apparatus

The Q500 Sonicator ultrasonic processor was purchased from Qsonica (Newtown, CT, USA). Milli-Q water was produced by using a PURELAB Flex 2 water purifier from ELGA LabWater (High Wycombe, UK). KrosFlo KR2i tangential flow filtration (TFF) equipment was purchased from Repligen (Waltham, MA, USA). Additionally, 96-well, clear bottom, high-binding polystyrene microtiter plates and Slide-A-Lyzer dialysis cassettes were purchased from Thermo Fisher Scientific (Waltham, MA, USA). 4%–20% Tris-glycine sulfate-polyacrylamide gel electrophoresis (SDS-PAGE) gels were purchased from Bio-Rad (Hercules, CA, USA). Novex IEF gels (pH 3–10) were purchased from Thermo Fisher Scientific (Waltham, MA, USA). The wash steps were performed using Molecular Devices (Sunnyvale, CA, USA) MultiWash+ plate washer using five cycles of 250 μl/well of wash buffer, employing a 10 s shaking step at the end of each cycle. Absorbance measurements were performed either on a SpectraMax 190 microplate reader from Molecular Devices (San Jose, CA, USA) or a CLARIOstar Optima UV/Vis spectrophotometer from BMG LABTECH (Ortenberg, Germany).

### 2.3 Animal models

Male ApoE homozygous knockout mice (strain B6.129P2-*APOE^TM1UNC^*/J; ApoE^−/−^) were obtained from The Jackson Laboratory (JAX stock #002052). The mice were maintained and bred under standard pathogen-free conditions. All animal experiments described were approved by the University of Miami Institutional Animal Care and Use Committee (IACUC) under Protocol 22-090. All procedures must conform to the guidelines from Directive 2010/63/EU of the European Parliament on the protection of animals used for scientific purposes or the NIH Guide for the Care and Use of Laboratory Animals. The atherosclerosis model was prepared by feeding ApoE^−/−^ mice a high-fat diet (HFD; 0.2% total cholesterol, saturated fat >60% total fat, and high sucrose) (TD.88137 Envigo) for 18 weeks after weaning. To perform endpoint examination experiments, euthanasia was performed via inhaled CO_2_ followed by cervical dislocation post-CO_2_ to ensure proper euthanasia.

### 2.4 Fluorescence and bioluminescence *in vivo* imaging system imaging

To track the location and biodistribution of infused *id*LFA-1 as well as *id*LFA-1-nanocarrier-coated murine MSCs, we conducted mouse whole-body *in vivo* imaging system (*IVIS*) scanning. To test infused *id*LFA-1, HFD-fed ApoE^−/−^ mice at 22 weeks old were separated into four groups (*n* = 5). VivoTag XL 680 (PerkinElmer #NEV11119) was used as a fluorescent dye to track *id*LFA1 and control murine serum albumin (MSA, Lee BioSolutions #101-50) distribution. Group 1: ApoE^−/−^ mice were injected intravenously (*i.v.*) via tail vein cannulation with 50 µg/mouse of *id*LFA-1-VivoTag XL 680. Group 2: ApoE^−/−^ mice were injected (*i.v.*) with 140 µg/mouse of MSA–VivoTag XL 680. Group 3: ApoE^−/−^ mice were injected (*i.v.*) with an equivalent volume of phosphate-buffered saline (PBS). Group 4: ApoE^−/−^ mice were injected (*i.v.*) with VivoTag XL 680 only. Thirty minutes post-infusion of various *id*LFA-1, MSA, or PBS, mice were anesthetized with 2.5% isoflurane according to our institutional animal protocol and underwent whole-body scan using the PerkinElmer Xenogen IVIS Spectrum System. After whole-body scanning, the mice were euthanized, and the aortas were harvested immediately for imaging and scanned using the IVIS spectrum system.

Similarly, to test infused *id*LFA-1-nanocarrier-coated mouse MSCs, mouse MSCs pre-transduced with Luc2^+^/lentivirus (Luc2^+^/MSCs) were coated or uncoated and injected into four groups of HFD-fed ApoE^−/−^ mice: Group1, *id*LFA-1-nanocarrier-coated MSCs; Group 2, MSA-nanocarrier-coated MSCs; Group 3, dendrimer-coated MSCs; Group 4, MSCs alone. MSCs were isolated from the bone marrow of ApoE mice, which were not fed with HFD and aged at 12–16 weeks old with mixed sex. The studies involving animal participants were reviewed and approved by the University of Miami IACUC under Protocol 22-090.

Fifteen minutes post-infusion via tail vein injection of various MSCs, mice were injected intraperitoneally (*i.p.*) with D-luciferin (150 m/kg). After an additional 15 min, mice were anesthetized with 2.5% isoflurane according to our institutional animal protocol and underwent a whole-body scan using the PerkinElmer Xenogen IVIS Spectrum System. After whole-body scanning, the mice were euthanized, and the aortas were harvested immediately for imaging and scanned using the IVIS spectrum system.

Photon counts of either fluorescence or bioluminescence signals were acquired and analyzed using Living Image version 4.7 software. Regions of interest (ROIs) were drawn sections in the defined area and quantified using the physical, calibrated unit “Radiant Efficiency [p/sec/cm^2^/sr]/[µW/cm^2^].” The Living Image software normalizes automatically for sensitivity differences resulting from different exposure times without any user input required when ROI values are expressed in a calibrated, physical unit.

### 2.5 Preparation for recombinant *id*LFA-1 and nanocarriers

The expression of the *id*LFA-1 has been performed, with minor modifications, as described (30). Briefly, a plasmid containing the I-domain DNA sequence, pET-11a/LFA-1 was transformed into competent *E. coli* BL21 cells. The cells were grown in 5.0 ml LB broth containing 100 µg/ml ampicillin. The next day, 300 ml of LB broth containing 100 µg/ml ampicillin was inoculated with 5.0 ml of the refreshed overnight cultures. To refresh, the overnight cultures were centrifuged at 5,000 × *g* for 10 min, the spent LB broth was discarded, and the cell pellet was resuspended in the same volume of fresh LB broth. The flasks were incubated at 37 °C, with shaking at 250 rpm, until the optical density of the culture at 600 nm (OD600) was approximately 0.8. Then, 300 µl of 1.0 M IPTG and 3.0 ml of 1.0 M sterile magnesium chloride were added to have final concentrations of 1.0 mM and 10.0 mM, respectively. The flask was incubated at room temperature for 3.0 h, with shaking at 250 rpm. The culture was then centrifuged at 8,000 × *g* for 15 min at 4 °C to harvest the bacterial cells expressing *id*LFA-1 in the form of inclusion bodies. The LB broth was discarded, and the cell pellet was resuspended in 30 ml of homogenization buffer (50 mM Tris buffer containing 1.0 mM DTT, 10 mM magnesium chloride, 2.0 mM EDTA, and 5.0 mM benzamidine hydrochloride at pH 8) and sonicated using 1 s pulses for 20 min at a 20% amplitude. After the sonication was completed, the cell suspension was centrifuged at 18,000 × *g* at 4 °C for 15 min. Then, the pellet was washed by resuspending in 30 ml of homogenization buffer and centrifuging at 18,000 × *g* at 4 °C for 15 min. This washing step was performed once more, followed by a final wash using deionized water. The pellet, which consisted of denatured *id*LFA-1 in the form of inclusion bodies, was solubilized in about 30 ml of denaturation buffer (50 mM Tris buffer containing 2.0 mM DTT, and 6 M guanidine hydrochloride at pH 8) by rotating at room temperature for 1 h. Then, the solution was centrifuged at 18,000 × *g* at 4 °C for 15 min to remove any insoluble matter. The solution was diluted in 200 ml cold DI water at 4 °C followed by rapidly diluting in 4.0 L of cold renaturation buffer (50 mM Tris buffer containing 1.0 mM DTT and 5 mM magnesium sulfate and 7% glycerol at pH 8) to refold the *id*LFA-1 protein into its functional form. This solution contains dilute, purified, *id*LFA-1 which was then concentrated using a 3,500 MWCO tangential flow filtration column. After concentrating the protein sample 40-fold, the sample was dialyzed using 10,000 MWCO dialysis cassettes into phosphate-buffered saline at pH 7.4. Any formed precipitate during dialysis was removed by centrifuging at 18,000 × *g* at 4 °C for 15 min. The purity of the protein was confirmed using sodium dodecyl sulfate polyacrylamide gel electrophoresis (SDS-PAGE), followed by the measurement of the protein concentration using a BCA assay. The isoelectric point of the purified protein was determined using Novex IEF gel (pH 3–10) according to the manufacturer's protocol.

### 2.6 Circular dichroism spectroscopy analysis

Samples of *id*LFA-1 were dialyzed into circular dichroism (CD) buffer containing 10 mM potassium phosphate and 100 mM ammonium sulfate at pH 7.40 at a final concentration of 0.1 mg/ml, and the far-UV CD spectra were recorded using a Jasco J-815 Spectropolarimeter (Jasco, Easton, MD, USA) [scan mode, continuous; scan speed 50 nm/min; data pitch 0.5 nm; bandwidth 1 nm; data integration time (DIT) 2 s; accumulations 3] (31). Analysis of the CD data was performed using DichroWeb (http://dichroweb.cryst.bbk.ac.uk), and secondary structure assignments were generated using the CDSSTR analysis package.

### 2.7 Labeling of *id*LFA-1, MSA, and BSA with fluorophores Cy5, and VivoTag XL 680 (VT-XL680)

The proteins used in this study were labeled with the fluorophores Cy5 and VT-XL680 according to the manufacturer's protocols. Briefly, an aliquot of 1.0 mg/ml of the protein, dissolved in 50 mM carbonate/bicarbonate buffer at pH 8, was reacted with the 4× mole excess of the fluorophore, dissolved at a concentration of 1.0 mg/ml in anhydrous DMSO, at room temperature for 2 h. The excess, unreacted fluorophore, was then removed by dialysis against PBS at pH 7.40, and the protein concentration and the fluorophore labeling efficiency were measured according to the manufacturer's protocol using a UV/Vis spectrophotometer.

### 2.8 Preparation of tagged protein–Ac-G5 nanocarriers and cell surface coating with nanocarriers

The nanocarriers were prepared by complexing 2× mole excess of commercially purchased 20% acetylated G5-PAMAM dendrimers with either labeled or unlabeled proteins. Briefly, to coat 1.0 × 10^6^ cells, 1.4 nanomoles of protein of interest and 2.8 nanomoles of 20% acetylated G5-PAMAM dendrimer was mixed at room temperature for 15 min. Both the dendrimer and the protein were dissolved in the Opti-MEM medium separately, and the dendrimer solution was added, dropwise, to the protein solution, while mixing. The mixture was then incubated at room temperature for 15 min to allow for the complexation. Any formed precipitate during incubation was removed by centrifuging at 18,000 × *g* at 4 °C for 15 min.

### 2.9 Preparation of MSCs coated with nanocarriers

To coat the MSCs, an aliquot of 1.0 ml of nanocarriers, prepared as described above, in the Opti-MEM medium was mixed with 1.0 × 10^6^ MSCs and incubated for 20 min at room temperature with gentle mixing every 5 min. Afterward, nanocarrier-coated MSCs were centrifuged at 270 × *g* for 5 min and gently resuspended with a pipette in 5.0 ml sterile PBS. As mentioned above, the studies involving ApoE^−/−^ mice were reviewed and approved by the University of Miami IACUC under Protocol 22-090.

### 2.10 Recombinant lentiviruses and cell transduction

DsRed/lentiviral vector plasmid, LacZ/lentiviral vector plasmid, and Luc2/lentiviral vector plasmid were described previously (5). Human ICAM-1/lentiviral vector was purchased from GenTarget Inc. (LVP595, San Diego, CA, USA). Production of pseudotyped lentivirus was achieved by co-transfecting 293T cells with three plasmids as described (10). 293T cells were obtained from ATCC (CRL-3216). The lentiviruses collected 48 h post-transfection displayed titers of approximately 10^7^ transducing units/ml as determined by PCR. Mouse bone marrow-derived MSCs (32) and human microvascular endothelial cells (HMVEC) (33), which were kindly provided by D. Fraker, University of Pennsylvania (Philadelphia), were prepared and cultured as previously described. The study related to HMVEC was conducted in accordance with the Declaration of Helsinki and approved by the Ethics Committee of the University of Miami. To infect cells by lentivirus, cells were exposed for 6 h to a virus with a multiplicity of infection (MOI) of five viral particles/cell in the presence of 4 μg/ml polybrene (Sigma-Aldrich). Cells were then washed, cultured with regular complete medium for two additional days, and analyzed by fluorescence microscopy (for DsRed^+^/MSCs) or immunoblotting (for ICAM-1/lentivirus-transduced HMVEC^ICAM−*1hi*^ and LacZ/lentivirus-transduced HMVEC^ICAM−1*lo*^) or bioluminescence reader (SpectraMax L, Molecular Devices) (for Luc2^+^/MSCs), using standard protocols (5). Cells were pooled for subsequent analysis as indicated in individual experiments.

### 2.11 *In vitro* binding assays

1 × 10^5^ cells/well of HMVEC^ICAM−1*hi*^ were cultured in the 24-well glass plates (P^11^24G-1.5-10-F, MatTek) pre-coated with 1% gelatin, and cells reached 100% confluence 1 day later. To test the binding of fluorescent dye-labeled *id*LFA-1 or *id*LFA-1 nanocarrier on HMVEC^ICAM−1*hi*^, 100 μM *id*LFA1-Cy5 vs BSA-Cy5 or *id*LFA1-Cy5-conjugated Ac-G5 nanocarrier vs BSA-Cy5-conjugated Ac-G5 nanocarrier was added to the well in which an HMVEC monolayer was formed. After incubation for 30 min at 37 °C, wells were washed with PBS twice, and Cy5 signals that remained in wells were visualized by fluorescence microscopy and quantified by fluorescence scanner (GE Typhoon Trio, Piscataway, NJ, USA). Similarly, to test binding *id*LFA-1-nanocarrier-coated cells on HMVEC^ICAM−1*hi*^, 1 × 10^5^ mouse bone marrow-derived MSCs pre-transduced with DsRed/lentivirus (DsRed^+^/MSCs), which were pre-coated with *id*LFA-1-Ac-G5 and BSA-Ac-G5 nanocarriers, were suspended in 1 ml basal MesenCult (STEMCELL Technologies, Cambridge, MA, USA) medium and added to the well in which a HMVEC monolayer was formed and incubated for 1 h at 37 °C. Unbounded MSCs were washed out twice with PBS. Red fluorescence signals derived from adherent DsRed^+^/MSCs were measured and quantified by fluorescence scanner. All *in vitro* binding assays were duplicated in 24-well plates, and experiments were repeated three times.

### 2.12 Immunofluorescence assays

Immunofluorescence studies were performed in tissue sections to evaluate the expression of ICAM-1 on endothelial cells in the aorta, homing of nanocarrier-coated MSCs to atherosclerotic lesions, and biodistribution of nanocarrier-coated MSCs in the lung and liver. Tissue section slides were deparaffinized per standard protocol, and antigen retrieval was performed in EDTA buffer (pH 9) at 100 °C for 10 min. Slides were then washed in distilled water and permeabilized with 0.25% Triton X-100 in TBS for 15 min and rinsed twice with PBS. Slides were subsequently incubated with Protein Block (X0909, Agilent Dako) for 1 h. To detect various proteins, the following antibodies were utilized: Alexa Fluor 488 anti-CD31 antibody (EPR17259, ab305267, Abcam), Alexa Fluor 647 anti-firefly luciferase antibody (EPR17790, ab233049, Abcam), Alexa Fluor® 488 E-selectin/CD62E antibody (103, NBP289430AF488, Novus Biologicals). Slides were incubated overnight at 4 °C with antibodies in the protein block for 1 h at room temperature. The slides were then washed with 0.1% Tris-buffered saline and Tween-20 (TBST) prior to 4′,6-diamidino-2-phenylindole dihydrochloride (DAPI) staining (D9542, Sigma-Aldrich) for nuclear visualization. Slides were imaged utilizing an Olympus IX71 inverted fluorescence microscope.

### 2.13 Statistical analyses

Analysis of statistical differences was performed utilizing ANOVA for multiple samples of more than three groups and two-tailed Student's *t*-test for pairwise comparison. Data are expressed as mean ± standard deviation (SD), and values are considered significant based on a threshold of *p* < 0.05. Individual data points are shown in bar graphs.

## 3 Results

### 3.1 ICAM-1 expression is elevated on endothelium in the aortas of ApoE^−/−^ mice with atherosclerosis

Atherosclerosis is a chronic vascular inflammatory disease. ICAM-1 is well known to play a role in the recruitment of immune cells to sites of inflammation, including atherosclerotic lesions. To establish the presence of ICAM-1 in atherosclerotic lesions in our experimental animal model, we conducted immunofluorescence analysis (IFA) to examine the expression of ICAM-1 on luminal endothelium in the aortas of 22-week-old ApoE^−/−^ mice fed with HFD to recapitulate atherosclerosis. IFA confirmed elevated expression of ICAM-1 colocalization with the luminal endothelial cell marker CD31 in the aortas of ApoE^−/−^ mice fed with HFD+. Elevated levels of ICAM-1 were observed on luminal endothelium, small vessels, and capillaries in tunica intima and adventitia (Supplementary Figure 1A). In contrast, expression of ICAM-1 was rarely detectable in the aortas of control ApoE^−/−^ mice fed with a standard diet (HFD−). Interestingly, levels of ICAM-1 were robustly higher in endothelium in plaques (Supplementary Figure 1B), indicating a correlation between vascular inflammation status and ICAM-1 expression. These results confirmed that ICAM-1 expression is elevated on inflamed endothelium in the aortas, especially in plaques, of ApoE^−/−^ mice with atherosclerosis.

### 3.2 Purification of *id*LFA-1 and verification of *id*LFA-1/ICAM-1 association

**Figure 1:**
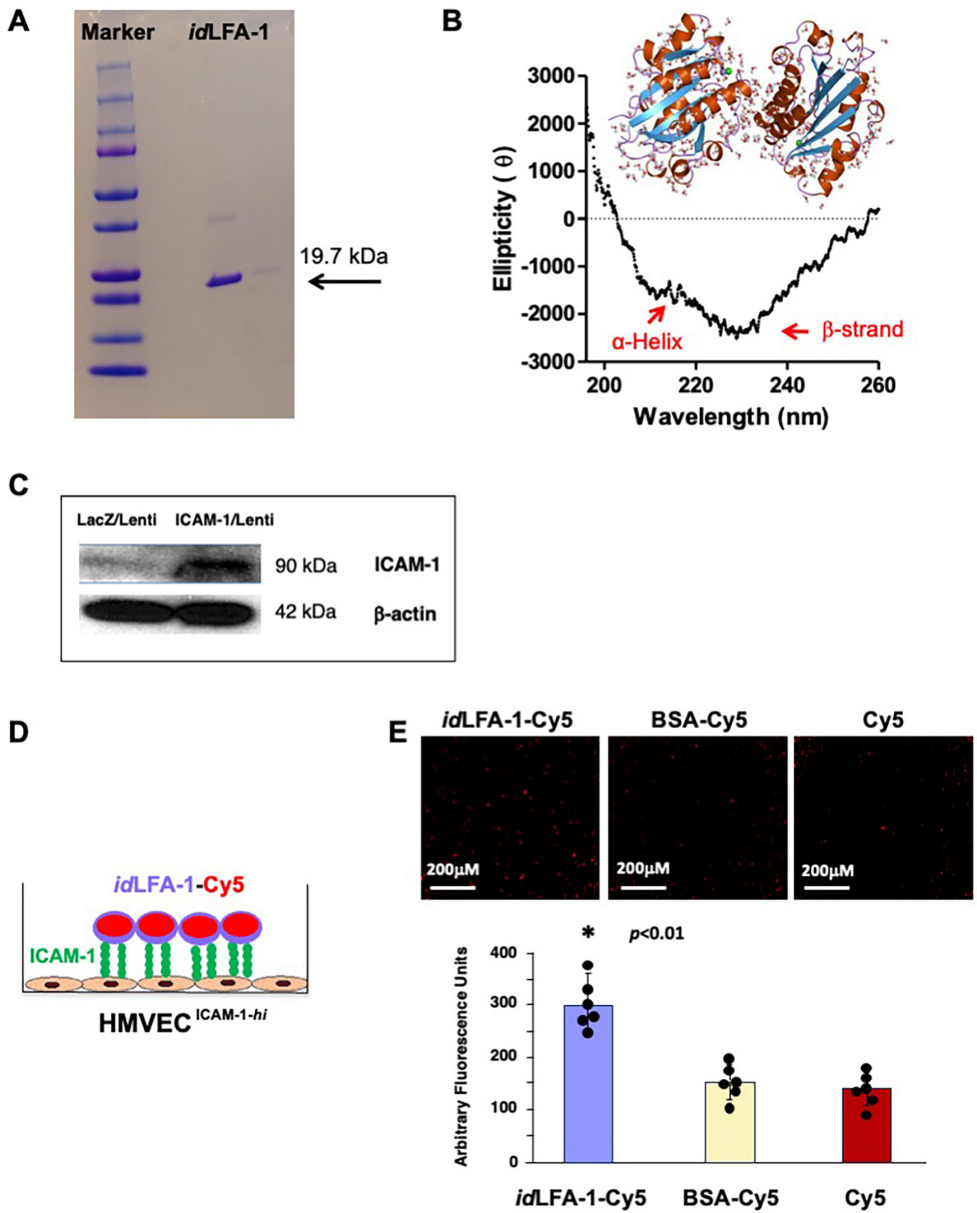
Verification of recombinant *id*LFA-1 purity and binding capability with ICAM-1 *in vitro*. **(A)** Recombinant purification of *id*LFA-1 with the appropriate molecular weight (19.7 kDa) was verified. **(B)** Circular dichroism spectra were collected to confirm the appropriate tertiary structure (α-helix and β-strand) of purified *id*LFA-1. **(C)** Western blot analysis demonstrating overexpression of ICAM-1 protein in HMVEC transduced with ICAM-1/lentivirus and control LacZ/lentivirus. **(D)** Schematic illustration of the *id*LFA-1-Cy5-(Ac-G5) dendrimer conjugate moiety binding to endothelial cells stimulated to express ICAM-1 (EC^ICAM−1−*hi*^). **(E)** Microscopy imaging demonstrating the association of *id*LFA-1-Cy5 with EC^ICAM−1−*hi*^ compared with BSA-Cy5-(Ac-G5) and Cy5-(Ac-G5) alone as well as quantitative data of Cy5 signal. Sizes of scale bars are shown. Data are presented as mean ± SD of three independent assays in which samples were duplicated. The asterisk denotes statistical significance (*p* < 0.01, ANOVA).

We sought to take advantage of ICAM-1/LFA-1 adhesion molecule pairs to mediate cell–cell interaction in the circulatory interface to achieve targeted cell delivery (directly homing the infused circulating *id*LFA-1-coated MSCs to the ICAM-1-expressing inflamed ECs on the lumen of the aorta). Since ICAM-1 expression is elevated on endothelium in the aortas of ApoE^−/−^ mice with atherosclerosis, we utilized the I-domain of LFA-1 (*id*LFA-1) as a payload to create nanocarriers given that I-domain (*id*) is responsible for mediating ICAM-1/LFA-1 interactions. In addition, using *id*, a smaller fragment of LFA-1, can increase the amount of payload in nanocarriers, yet decrease cost and potential side effects caused by large/whole LFA-1 protein. Nanocarrier systems typically comprise nanoparticles (dendrimers, PLGA, liposomes) and payloads, such as the target agent or protein moiety, to form vehicles to guide the cells to the proper location. The *id* of LFA-1 was overexpressed and purified from *E. coli* cells, in the form of inclusion bodies. The inclusion bodies have native-like secondary structures of the expressed protein, and they are resistant to proteolytic degradation. Since they are formed due to specific molecular interactions among a single type of protein, they mostly consist of the target recombinant protein in relatively high purity (33). Therefore, our purification strategy to obtain pure *id*LFA-1 involved isolating the formed inclusion bodies of *id*LFA-1 and extensive washing of the protein pellet, followed by a denaturation and renaturation cycle as described by Manikwar et al. (24). The purified protein was then characterized for its purity. In SDS-PAGE gel, the purified protein runs as a single band with an apparent molecular weight of 19.7 kDa (Figure 1A). The protein was also characterized with respect to its tertiary structure using circular dichroism (CD) spectroscopy. The purified *id*LFA-1 protein was consistent with the expected *id*LFA-1 characteristics (Figure 1B). To test the binding activity of *id*LFA-1, we carried out a cell binding assay using *id*LFA-1 conjugated to the fluorescent probe Cy5 for visualization and quantification by fluorescence microscopy and scanner, using a previously described method (5).

Overexpression of ICAM-1 was achieved by transduction of HMVEC with ICAM-1/lentivirus vs LacZ/lentivirus (control). Levels of ICAM-1 in HMVEC^ICAM−1*hi*^ vs HMVEC^ICAM−1*lo*^ was confirmed by immunoblotting (Figure 1C) and confirmed to be 16-fold. Figure 1D illustrates how this *in vitro* binding assay is performed. HMVEC^ICAM−1*hi*^ was cultured in 24-well glass plates to form a cell monolayer. A concentration of 100 μM *id*LFA1-Cy5 (test) vs BSA-Cy5 (control) vs Cy5 (blank) was added to wells in which monolayers of HMVEC^ICAM−1*hi*^ had been established. After incubation for 30 min at 37 °C, wells were washed with PBS twice, and Cy5 signals remaining in wells were visualized by microscopy and quantified by fluorescence scanner. We observed significantly higher Cy5 signal remained in wells added with *id*LFA1-Cy5 compared with wells exposed to BSA-Cy5 and Cy5 alone (Figure 1E). These results demonstrated that purified *id*LFA-1 fragments carry the anticipated biological activity and can bind with ICAM-1 on the cell surface.

### 3.3 Binding of *id*LFA-1-nanocarriers and *id*LFA-1-nanocarrier-coated MSCs with ICAM-1 *in vitro*

**Figure 2:**
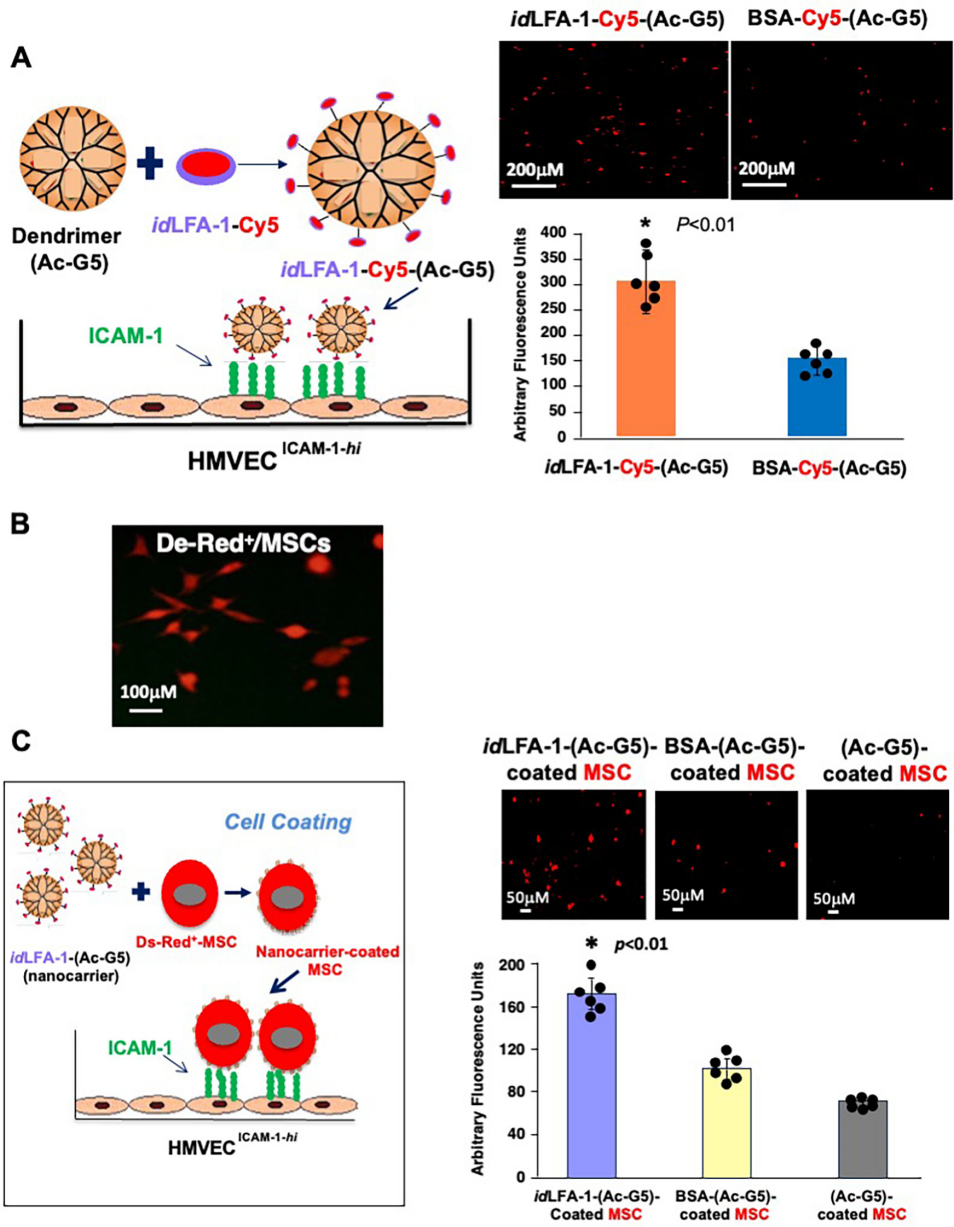
*id*LFA-1-Cy5-(Ac-G5) dendrimer nanocarriers and *id*LFA-1-Cy5-(Ac-G5) nanocarrier-coated MSCs preferentially bind to ICAM-1 expressing endothelial cells *in vitro*. **(A)** *Left*: Schematic illustration of the *id*LFA-1-Cy5-(Ac-G5) dendrimer conjugate moiety binding to HMVEC transduced with ICAM-1/lentivirus to overexpress ICAM-1 (HMVEC^ICAM−1−*hi*^). *Right*: Fluorescent microscopy imaging demonstrating an increased association of *id*LFA-1-Cy5-(Ac-G5) with HMVEC^ICAM−1−*hi*^ compared with BSA-Cy5-(Ac-G5). **(B)** Fluorescent microscopy demonstrating DsRed fluorescent expression in MSCs after transduction with DsRed/lentivirus. **(C)** *Left*: Schematic illustration of binding of *id*LFA-1-Cy5-(Ac-G5)-coated MSCs with HMVEC^ICAM−1−*hi*^. *Right*: Fluorescent microscopy imaging demonstrating the association of *id*LFA-1-(Ac-G5)-coated MSCs binding with HMVEC^ICAM−1−*hi*^ compared BSA-nanocarrier- and nanocarrier-alone-coated MSCs. Quantitative data of Cy5 signal. Data are presented as mean ± SD of three independent assays in which samples were duplicated. The asterisk denotes statistical significance (*p < 0.01, **A**: Student's *t*-test, **C**: ANOVA). Sizes of scale bars are shown.

We next sought to validate the binding capability between *id*LFA-1-Cy5-(Ac-G5) dendrimer nanocarriers and endothelial cells expressing ICAM-1. We specifically investigated the interaction between *id*LFA-1-Cy5-(Ac-G5) and HMVEC^ICAM−1*hi*^. For this, the *id*LFA-1-Cy5 was complexed to Ac-G5 dendrimer to form nanocarriers [*id*LFA-1-Cy5-(Ac-G5)]. BSA nanocarriers [BSA-Cy5-(Ac-G5)] were constructed as control. The *id*LFA-1-Cy5-(Ac-G5) and BSA-Cy5-(Ac-G5) were added to wells in which monolayers of HMVEC^ICAM−1*hi*^ were present (Figure 2A, *left*). After incubation for 30 min at 37 °C, wells were washed with PBS twice, and the remaining Cy5 signals in wells were visualized by fluorescence microscopy and quantified by employing a fluorescence scanner. Through these experiments and by employing fluorescent microscopy, we found that *id*LFA-1-Cy5-(Ac-G5) exhibited superior adhesion to the HMVEC^ICAM−1*hi*^ monolayers compared with BSA-Cy5-(Ac-G5) (Figure 2A, *right*). This further demonstrated that *id*LFA-1 nanocarriers can bind to ICAM-1 on the surface of ECs.

We next tested whether *id*LFA-1-nanocarrier-coated MSCs were able to bind to ICAM-1 on the surface of ECs. Mouse bone marrow-derived MSCs, pre-transduced with DsRed/lentivirus (DsRed^+^/MSCs) (Figure 2B), were coated with *id*LFA-1-(Ac-G5) nanocarriers, and controls included BSA-Cy5-(Ac-G5)- and Cy5-(Ac-G5) nanocarrier-coated MSCs. Similarly, three nanocarrier-coated MSC groups [*id*LFA-1-Cy5-(Ac-G5)-, BSA-Cy5-(Ac-G5)- and Cy5-(Ac-G5)-coated MSCs] were added to wells in which monolayers of HMVEC^ICAM−1*hi*^ were seeded as illustrated in Figure 2C, *left*. After incubation for 30 min at 37 °C, wells were washed with PBS twice, and DsRed^+^/MSCs associated with HMVEC^ICAM−1*hi*^ in wells were visualized by fluorescence microscopy and quantified by using a fluorescence scanner. We determined that there were significantly higher numbers of *id*LFA-1-Cy5-(Ac-G5)-coated MSCs associated with HMVEC^ICAM−1*hi*^ compared with BSA-Cy5-(Ac-G5) and Cy5-(Ac-G5) nanocarrier-coated MSCs (Figure 2C, *right*). Taken together, our data demonstrated that *id*LFA-1 can mediate cell–cell interaction in an *in vitro* model.

### 3.4 *id*LFA-1 binds to atherosclerotic lesions in the aorta of an ApoE^−/−^ mouse

**Figure 3:**
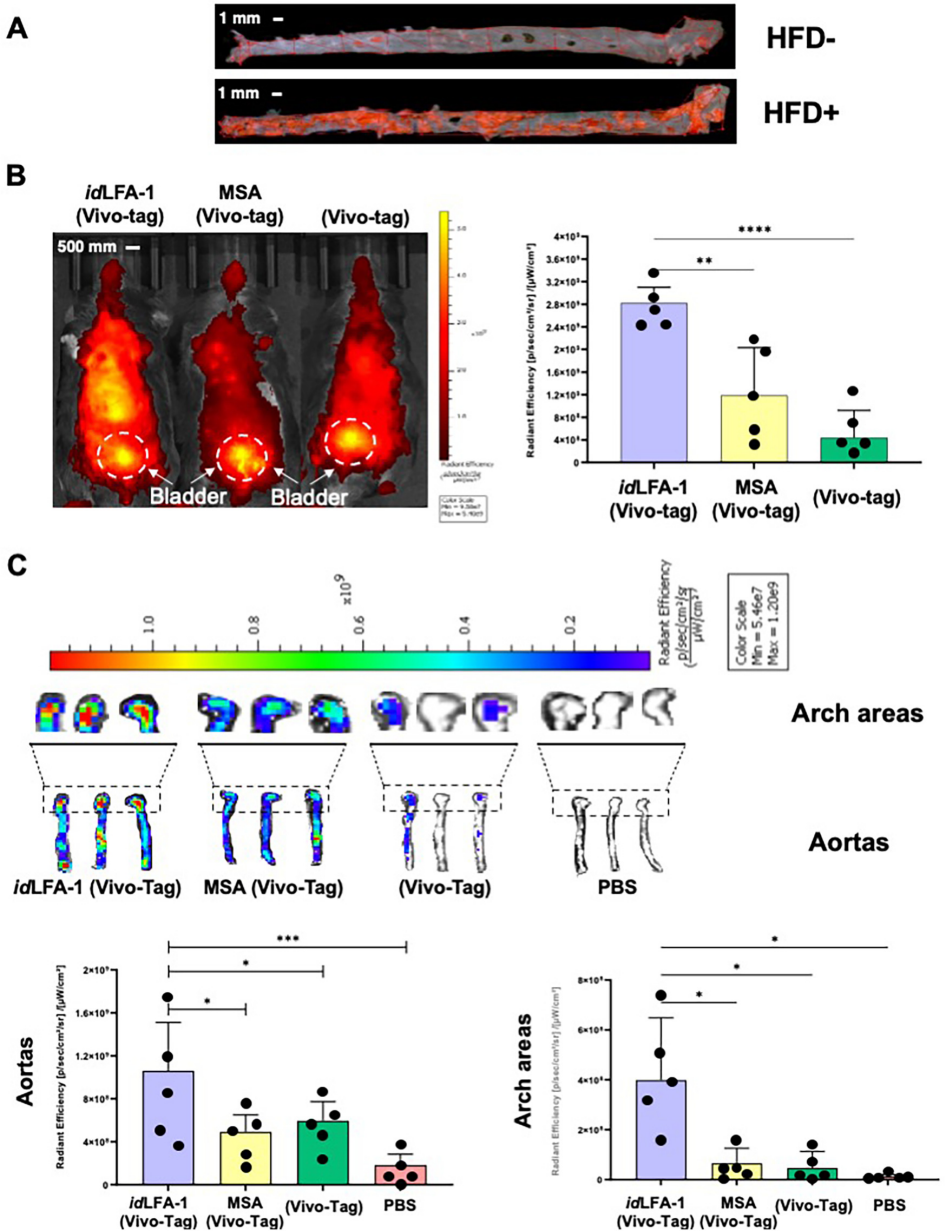
Targeted delivery of *id*LFA-1 to the aorta and aortic arch after systemic infusion *in vivo.* **(A)** Whole aorta visualization of atherosclerotic plaques by Oil Red O staining in ApoE^−/−^ mice fed with or without HFD. **(B)** *Left*: *IVIS* imaging shows an increased signal intensity of *id*LFA-1 conjugated to VivoTag dye throughout the body and most dense in the midline compared with murine serum albumin (MSA)–VivoTag and VivoTag controls 30 min after systemic injection in ApoE^−/−^ mice fed with HFD. *Right*: Quantitative data of VivoTag fluorescent dye intensity in each group of mice (*n* = 5/group). The asterisks denote statistical significance (**p = 0.022; ****p < 0.0001, ANOVA for multi-samples and Student's *t*-test for pairwise comparison). **(C)** *Top*: Three representative *IVIS* imaging shows an increased uptake of *id*LFA-1 conjugated to VivoTag in the aortic arch areas and aortas harvested from ApoE^−/−^ mice fed with HFD compared with MSA–VivoTag, VivoTag alone, and PBS controls. *Bottom*: Quantification data of fluorescent dye signal intensity demonstrates higher signal in the aortas and arch areas of mice receiving *id*LFA-1 conjugated to VivoTag. The asterisks denote statistical significance (*p < 0.05; ***p < 0.001, ANOVA for multi-samples and Student's *t*-test for pairwise comparison, *n* = 5/group). Sizes of scale bars are shown.

We then tested the ability of *id*LFA-1 to bind to inflamed atherosclerotic lesions in the aorta of an ApoE^−/−^ mouse fed with HFD. The *id*LFA-1 was conjugated to a fluorochrome (VivoTag XL680, PerkinElmer #NEV11119) for *in vivo* IVIS imaging. Equal volumes of 100 μl of VivoTag–*id*LFA-1, VivoTag–MSA (murine serum albumin, Lee BioSolutions #101-50), and VivoTag alone were intravascularly injected into 22-week-old ApoE^−/−^ mice fed with HFD via tail vein (*i.v.*) (five mice/group). Development of atherosclerosis in the aortas of ApoE^−/−^ mice was pre-demonstrated by *Oil Red O* staining (Figure 3A). Mice were subjected to a whole-body IVIS scan for 30 min following *i.v.* injection. Compared to control mice injected with VivoTag–MSA (1.19 ± 0.84 × 10^9^ [p/s/cm^2^/sr]/[µW/cm^2^]) and VivoTag alone (4.42 ± 4.80 × 10^8^ [p/s/cm^2^/sr]/[µW/cm^2^]), a significantly higher amount of VivoTag–*id*LFA-1 was present in the region of the aorta (2.82 ± 0.28 × 10^9^ [p/s/cm^2^/sr]/[µW/cm^2^]) (Figure 3B). VivoTag signals in the bladders of all three groups of mice are comparable, indicating that equal amounts of VivoTag–*id*LFA-1, VivoTag–MSA, and VivoTag were injected into mice. The signals in the region of the aorta were normalized by those in the bladder. After a whole-body scan, the aortas were immediately resected and subjected to an IVIS scan. Consistent with the results of the whole-body scan, a significantly higher amount of VivoTag–*id*LFA-1 signal was detected in the aortas of mice injected with VivoTag–*id*LFA-1 (1.06 ± 0.50 × 10^9^ [p/s/cm^2^/sr]/[µW/cm^2^]) compared with control mice injected with VivoTag–MSA (4.91 ± 1.77 × 10^8^ [p/s/cm^2^/sr]/[µW/cm^2^]) and VivoTag alone (5.47 ± 1.74 × 10^8^ [p/s/cm^2^/sr]/[µW/cm^2^]) (Figure 3C). Because VivoTag–*id*LFA-1 signals were richer in arch regions of the aorta where mice have more severe atherosclerotic lesions, we also compared the intensity of the VivoTag–*id*LFA-1 signals in arch regions. Consistent with results of the whole aorta, a robustly increased VivoTag–*id*LFA-1 signal intensity was presented in the arch of the aorta in mice injected with VivoTag–*id*LFA-1 (3.99 ± 2.50 × 10^8^ [p/s/cm^2^/sr]/[µW/cm^2^]) compared with control mice injected with VivoTag–MSA (6.57 ± 6.02 × 10^7^ [p/s/cm^2^/sr]/[µW/cm^2^]) and VivoTag alone (4.66 ± 6.61 × 10^7^ [p/s/cm^2^/sr]/[µW/cm^2^]). Taken together, these results demonstrated that infused circulating *id*LFA-1 can home and bind to atherosclerotic lesions in the aortas of ApoE^−/−^ mice, indicating that *id*LFA-1 can be used as targeting moiety to create nanocarriers to mediate targeted cell delivery to atherosclerotic lesions *in vivo*.

### 3.5 *id*LFA-1-nanocarrier-coated MSCs home to atherosclerotic aorta lesions in ApoE^−/−^ mouse model

**Figure 4:**
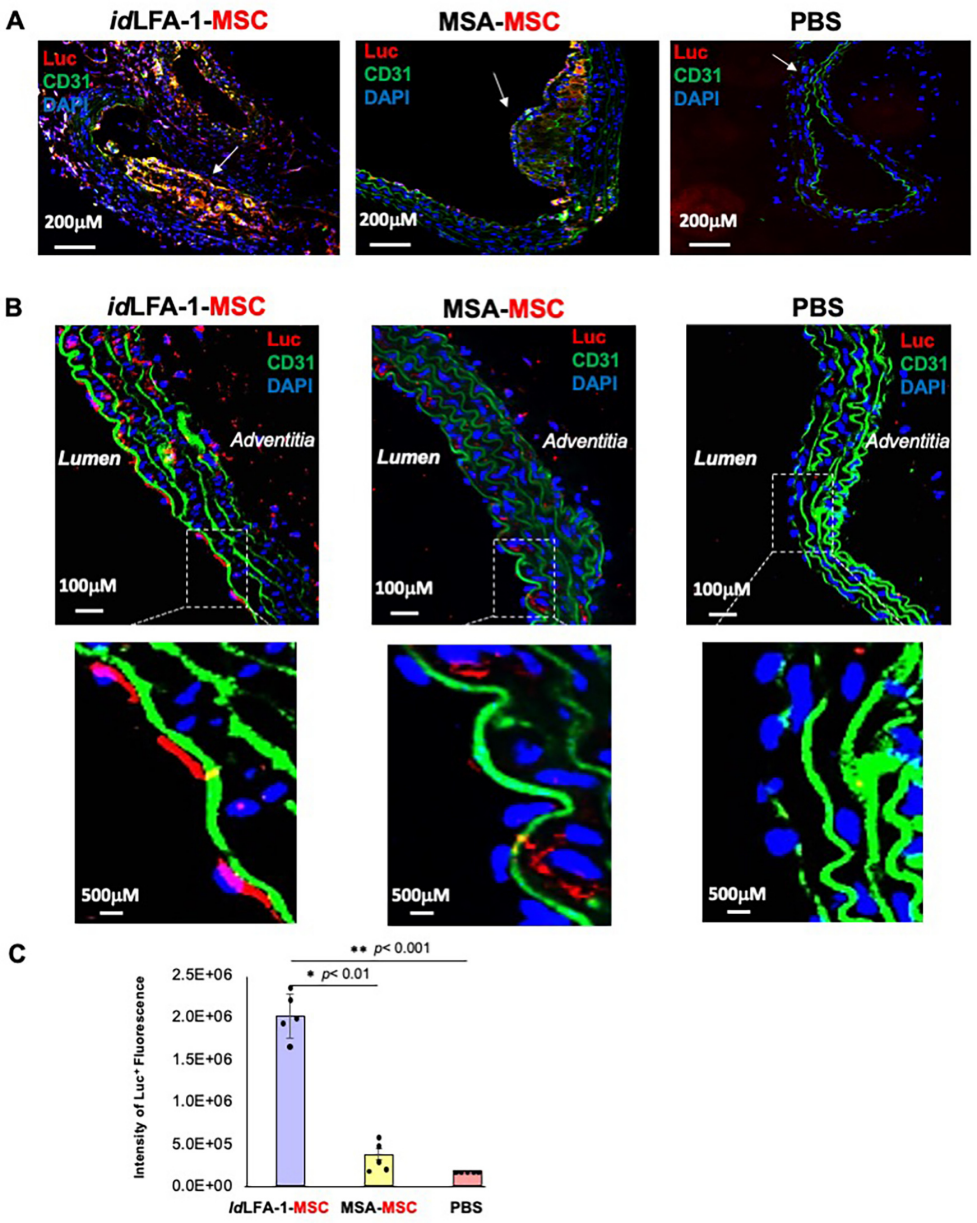
Targeted delivery of *id*LFA-1-nanocarrier-coated MSCs to the inflamed aortas in ApoE^−/−^ mice. **(A)** Representative immunofluorescent microscopy imaging shows increased colocalization (orange and purple) of *id*LFA-1-coated Luc^+^ MSCs (red) with nuclei (blue) at aortic plaque sites of aortas (white arrows) harvested from ApoE^−/−^ mice fed with HFD 30 min after luciferin injection compared with PBS and murine serum albumin-coated MSC controls. **(B)** Representative immunofluorescent microscopy imaging shows increased binding of *id*LFA-1-coated Luc^+^ MSCs (red) with nuclei (blue) at the lumen in non-plaque areas of aortas harvested from ApoE^−/−^ mice fed with HFD 30 min after luciferin injection compared with PBS and murine serum albumin-coated MSC controls. Higher magnification images demonstrating Luc^+^ MSCs (red) attachment at the luminal endothelium (green, along with strong green autofluorescence from elastin). **(C)** Quantitative data of fluorescent images of Luc^+^ MSC signal. The asterisks denote statistical significance (*p < 0.01, **p < 0.001, ANOVA for multi-samples and Student's *t*-test for pairwise comparison). Sizes of scale bars are shown.

Next, we employed *id*LFA-1 as a targeting moiety to create nanocarriers for targeted delivery of MSCs to atherosclerotic lesions *in vivo*. The *id*LFA-1-nanocarriers [*id*LFA-1-(Ac-G5)] and control nanocarriers [MSA-(Ac-G5)] were created and applied to coat mouse MSCs, which were pre-transduced with Luc2/lentivirus as described above. A total of 1 × 10^6^ Luc2^+^/MSCs coated with *id*LFA-1-(Ac-G5) vs MSA-(Ac-G5) were suspended in 100 μl of PBS and intravascularly injected to 22-week-old ApoE^−/−^ mice fed with HFD via tail vein (*i.v.*) (five mice/group). Mice injected with 100 μl of PBS were used as a negative control. Fifteen minutes post cell infusion, mice were injected (*i.p.*) with D-luciferin. After an additional 15 min time elapsed, mice were sacrificed, and the aortas (and major organs, including the lung and liver; see biodistribution below) were harvested and subjected to IFA to detect homing of infused Luc2^+^/MSCs to atherosclerotic aorta lesions in ApoE^−/−^ mice. We observed that significantly more Luc2^+^/MSCs presented in the aortas of ApoE^−/−^ mice receiving *id*LFA-1-(Ac-G5)-MSCs compared with those treated with MSA-(Ac-G5)-MSCs. Many of Luc^+^/MSCs were enriched in the plaques where ICAM-1 levels are higher (Figure 4A). This is consistent with the levels of ICAM-1 that are robustly expressed in endothelium covering or within plaques. We also observed the homing of some Luc2^+^/MSCs to the lumen where there is an absence of plaques, but ICAM-1 levels are also elevated (Figure 4B). Luc2^+^ *id*LFA-1-(Ac-G5)–MSCs were detectable in the lumen of the aorta and small vessels and capillaries in the tunica intima and adventitia. In contrast, significantly lower numbers of Luc2^+^ MSA-(Ac-G5)–MSCs were detectable in the aortas of control ApoE^−/−^ mice. No Luc2^+^ cells could be detectable in the aortas of ApoE^−/−^ control mice injected with PBS. Imaging with higher magnification revealed the attachment of Luc2^+^/MSCs delivered by *id*LFA-1-(Ac-G5) nanocarriers to the lumen of the aorta. Quantitative data are shown in Figure 4C. Our data demonstrated that *id*LFA-1-(Ac-G5) nanocarriers can successfully direct circulating MSCs home to inflamed endothelium at atherosclerotic aorta lesions of ApoE^−/−^ mice.

### 3.6 Biodistribution of *id*LFA-1 and *id*LFA-1-nanocarrier-coated MSCs in ApoE^−/−^ mouse model

**Figure 5:**
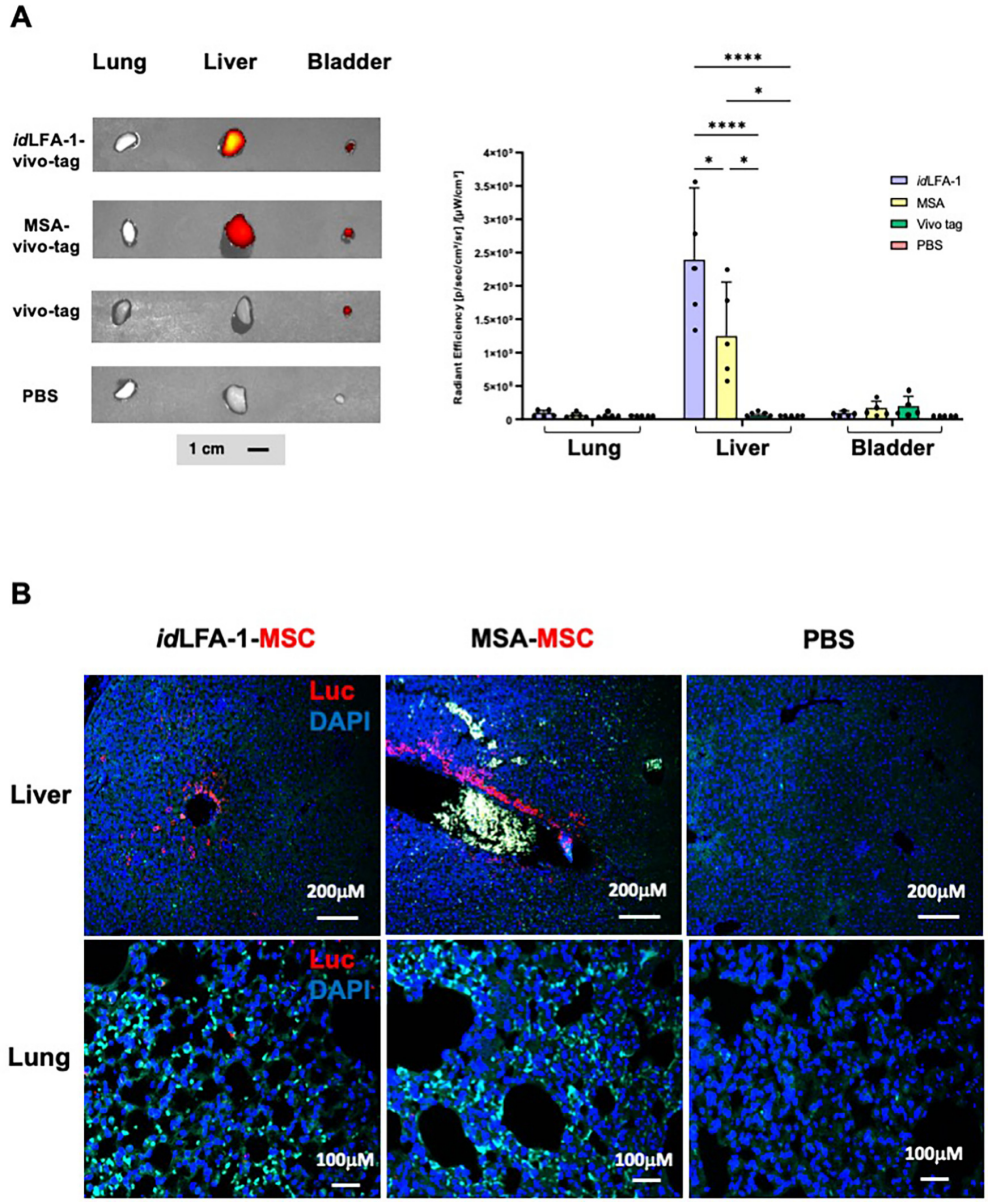
Biodistribution of *id*LFA-1 and *id*LFA-1-nanocarrier-coated MSCs. **(A)** *Left*: Fluorescent dye signal intensity showing *id*LFA-1–VivoTag homing to the liver without homing to the lung. The VivoTag signals detectable in the bladder are shown as a systemic loading control. *Right*: Quantitative data of fluorescent images of VivoTag signals. The asterisks denote statistical significance (*p < 0.05, ****p < 0.001, ANOVA for multi-samples and Student's *t*-test for pairwise comparison, *n* = 5/group). **(B)** Representative immunofluorescent microscopy imaging shows comparable deposition of *id*LFA-1-coated Luc^+^ MSCs (red) with nuclei (blue) at hepatic sinusoids harvested 30 min after systemic injection from ApoE^−/−^ mice fed with HFD as that injected with murine serum albumin-coated MSCs. Green signals are autofluorescence from some blood cells. Imaging of tissues from mice injected with PBS is used as baseline control. Sizes of scale bars are shown.

Next, we addressed how specifically *id*LFA-1–VivoTag XL680 can traffic and bind on atherosclerotic aorta lesions. Whole-body IVIS scan imaging did not detect that infused *id*LFA-1–VivoTag XL680 are enriched in any major organs except in the region of the aortas (see Figure 3B), suggesting that *id*LFA-1 can specifically bind to inflamed endothelium rather than normal endothelium. Immediately following whole-body scanning, the lungs, livers, and bladders of the animals were excised and subjected to an IVIS scan. No obvious signals were detectable in the lungs harvested from four groups of ApoE^−/−^ mice injected with *id*LFA-1–VivoTag XL680, MSA–VivoTag XL680, PBS, and VivoTag XL680 alone, respectively (Figure 5A). Although some VivoTag XL680 signal was detected in the livers of ApoE^−/−^ mice injected with *id*LFA-1–VivoTag XL680 and MSA–VivoTag XL680, there was no significant difference in the intensity of VivoTag XL680 signals between these two groups of livers (Figure 5A, *right*), thus indicating that these signals were not due to specific binding of *id*LFA-1 or MSA to the liver. This may be due in part to partially inflamed endothelium in the livers of ApoE^−/−^ mice induced by the HFD.

To further test the biodistribution of *id*LFA-1-nanocarrier-coated MSCs vs MSA-nanocarrier-coated MSCs, we conducted immunostaining studies using anti-Luc antibodies to examine potential localization of Luc2^+^/MSC coated with of *id*LFA-1 nanocarrier or MSA nanocarrier in the lungs and livers of ApoE^−/−^ mice. Similar as shown in Figure 5A, no obvious Luc2^+^ MSCs were detectable in the lungs (Figure 5B, *bottom*). There were some Luc2^+^ MSCs present in the surroundings of hepatic vessels; however, there was no significant difference between these two groups of livers (Figure 5B, *top*), suggesting that these MSCs are not specifically directed to the liver by either the *id*LFA-1 nanocarrier or the MSA nanocarrier. This may be partially due to the high vessel density in hepatic tissue where infused circulating *id*LFA-1 or MSA can traffic and temporarily locate and enrich or it could be attributed to HFD-induced hepatic inflammation, which could lead to binding of the nanocarrier to the liver. These data suggest that *id*LFA-1 nanocarriers can selectively direct MSCs to home to the inflamed endothelium at atherosclerotic aorta lesions of ApoE^−/−^ mice.

## 4 Discussion

This work demonstrates an innovative utilization of an adhesion molecule moiety to design nanocarriers for cell surface coating such as to direct the injected cells to home specifically to diseased tissue. This cell delivery system mediated by modified nanocarriers offers superior specificity compared with conventional stem cell delivery with a significantly greater number of cells delivered to target tissues. By exploiting the relationship between a highly or selectively expressed adhesion molecule on luminal endothelial cells and its cognate adhesion moiety in pathological states such as atherosclerosis, therapeutic cells can discriminately traffic to target disease areas upon systemic injection. Here, we identified ICAM-1 to be highly expressed on endothelium in sites with atherosclerotic plaque burden and created nanocarriers with *id*LFA-1 to potentiate MSC delivery to diseased areas. This is the first demonstration of this nanocarrier-based method of targeted delivery to atherosclerotic plaque by employing the highly selective interaction between *id*LFA-1 ICAM-1. It should be noted that interactions between ligand and receptor can result in unintended effects including signal transduction and proinflammatory stimulation in endothelial cells. Thus, it is critical to carefully select specific moieties/adhesion domains for complexation with nanocarriers to result in maximum binding capacity without complete induction of unwanted intracellular cell signaling. In that regard, our method to install specific moieties/adhesion domains on the cell surface via nanocarrier can avoid potential unintended consequences of intracellular cell signaling initiated by interactions between ligand and receptor, which may induce uncontrolled cell differentiation, particularly when infusing MSCs and any other types of stem/progenitor cells.

Our nanocarrier-coated cell delivery technology platform employs the anionic properties of the cell membrane to associate with nanocarriers composed of cationic dendrimers. This ionic bond results in cell surface electrostatic attachment of nanocarrier–dendrimer complexes via complexation. Given the reversible, non-covalent relationship of this complexation, nanocarriers may theoretically disassociate with MSCs after injection and bond with other cell types *in vivo*. Despite this, our data indicate that nanocarrier-coated cells administered systemically maintain the ability to selectively migrate to target tissues, thereby demonstrating the residual high fidelity of these nanocarrier complexes on MSCs after injection. Other associations between nanocarriers and cell surface substrates such as protein or carbohydrates as well as incomplete internalization of nanocarriers after the initial coalescence of nanocarriers on the cell membrane could also explain this observed phenomenon. Another benefit of dendrimer–nanocarrier cell membrane coating is that the eventual complete internalization of these complexes by cells allows for non-toxic elimination from the body and, subsequently, results in enhanced biocompatibility. Given that this platform relies on physical non-covalent interactions of adhesion molecules on the cell surface without manipulation of cell populations underlying genetic expression or protein machinery, it can be tailored to incorporate nearly any adhesion moiety/ligand on any cell to program targeted delivery. Installation of *id*LFA-1 on MSCs via a non-genetic approach can avoid potential biosafety concerns related to some genetic elements required for gene delivery and regulation. The physical decoration of MSCs with nanocarriers has the added advantage of producing augmented homing to disease sites while preserving the native functional capacity of unmodified MSCs without other phenotypic or genetic alterations incurred by genetic or inducible expression of protein moieties with viral vectors. For example, we have previously demonstrated the role of an inducible cell adhesion molecule, E-selectin, in mediating endothelial progenitor cell recruitment to wound sites (5). By cell surface coating of MSCs with Ac-G5-sE-sel dendrimer nanocarriers, systemic cell delivery of MSCs to wounded tissue was substantially increased. This resulted in direct biological responses through both accelerated rates of wound closure and pro-angiogenic effects of MSCs through enhanced neovascularization in wound bed tissue (5).

Importantly, we further demonstrated the efficacy of these nanocarriers in enhancing *id*LFA-1/ICAM-1 interactions at three different levels: (1) *id*LFA-1/ICAM-1 *in vitro* protein–EC cell binding, (2) *id*LFA-1-Cy5-(Ac-G5)/ICAM-1 nanocarrier-EC cell binding *in vitro*, and (3) MSC-EC binding via *id*LFA-1-Cy5-(Ac-G5)/ICAM-1 both *in vitro* and *in vivo*. Previous studies demonstrated the utility of *id*LFA-1 as a strategy to deliver drug and/or target molecules to different cell types expressing surface ICAM-1 (24, 34). Others further demonstrated the ability of modified nanoparticles to deliver drug cargo to ICAM-1 expressing cell types for localization to target cell phenotypes (34). However, the manipulation of nanocarrier particles with *id*LFA-1 to improve the delivery of cell therapy has not been previously investigated. Given the specific homing of coated MSCs to luminal endothelium in atherosclerotic lesions, this suggests that ICAM-1 is present at high enough levels to recruit circulating cells at disease sites, even in locations subjected to the high velocity/dynamic circulatory flow of the aorta. Furthermore, the fact that BSA-nanocarrier-coated MSCs achieved only partial localization to sites of atherosclerosis demonstrates that upregulation of ICAM-1 levels alone is not sufficient to recruit unmodified MSCs into the aorta. Given that nanocarrier-coated MSCs were administered systemically, it is conceivable that these cells could also traffic via capillaries and postcapillary venules to alternative disease sites at peripheral locations that also express the target binding molecule of interest. As with any systemic administration method, there is the potential for indiscriminate trafficking to other organs. In particular, the lung and liver can decrease the effectiveness of cell engraftment given their dense capillary networks, which typically persist as cell filtration obstacles to conventional MSC therapies infused via venous routes (1). We specifically examined the lung and liver, two major organs that act as filter sinks collecting blood from systemic circulation veins (lung) and arteries (liver), and bladders that collect/store expelling *id*LFA-1 (*id*LFA-1–VivoTag XL680), thus serving as loading control. Interestingly, there was no significant signal intensity detected in *ex vivo* lung tissue. However, there was partial colocalization of *id*LFA-1-coated MSCs to hepatic vessels after systemic administration in our study. This can be caused in part by hepatic lipid elevation-induced inflammation, which is well known to occur in ApoE^−/−^ mice fed with HFD (35, 36). Excessive intake of fat can result in hepatic lipid accumulation, thereby leading to non-alcoholic fatty liver disease (NAFLD). NAFLD livers exhibit inflammation due to the infiltration of inflammatory macrophages and other myeloid cells in the hepatic parenchymal area (37). NAFLD-induced hepatic inflammation can further result in the upregulation of ICAM-1 on the endothelium within fatty liver tissues (38). Despite partial deposition of MSCs in the liver, there was still a significantly greater amount of *id*LFA-1-coated MSCs delivered to inflamed aortic endothelium. In addition to NAFLD-induced hepatic inflammation, partial deposition of MSCs in the liver could also be ascribed to hepatic clearance, which is known to occur in other MSC therapies (1, 39). Compared to biodistribution in hepatic tissue, we observed similar amounts of MSA-nanocarrier-coated MSCs present in the surrounding area of hepatic vessels/sinusoids. This may suggest that the deposition of MSCs in the liver can be due to a general indiscriminate clearance of MSC therapy from the body.

MSC therapy has shown promise in preclinical studies for reducing atherosclerotic burden and plaque progression given their numerous beneficial immunomodulatory effects. Transplantation of MSCs is associated with an increase in anti-inflammatory cytokines such as IL-10 and TGF-β, as well as attenuation of the release of inflammatory mediators such as TNF-α and NF-κB (40–43). A limitation of this work was the inability to examine the therapeutic effect of MSC therapy upon atherosclerotic lesions as our construct employed the human *id*LFA-1, which is immunogenic to experimental mice. Furthermore, our study was limited by the inability to assess the viability of cells homed to atherosclerotic regions. This will be the subject of future studies as well as examining key molecular determinants of MSC function and gene expression associated with engrafted cells. Subsequent work should take into consideration the design and homology of target proteins of interest in inducing maximum immunotolerance across preclinical *in vivo* models and translational clinical studies. An advantage of the described method is the ability to customize nanocarriers to enable direct delivery of various cell types to different tissue types, thus highlighting the significant potential as a versatile platform for cell-based therapeutics for a myriad of diseases. Finally, this approach can have broad utility and applications for cell-based therapy in transplantation, cardiovascular disease, autoimmune pathologies, and regenerative medicine.

## 5 Conclusions

We describe a novel application of newly designed nanocarriers for cell surface coating to guide the homing of systemic administrated circulating cells to disease sites. Specific cell adhesion moieties/molecule-tailored nanocarriers can act as a “GPS” and guide coated cells to find their destination via targeting the blood/vessel interface for cell delivery. The ability to customize this nanocarrier technology based on specific adhesion moieties/molecules and their cognate binding counterpart highly or selectively expressed on the endothelium in diseased tissue enables this as a highly versatile platform to enhance systemic delivery of cell-based therapeutics for a broad variety of clinically relevant translational purposes.

## Data Availability

The raw data supporting the conclusions of this article will be made available by the authors, without undue reservation.

## Supplementary material

Supplementary Figure 1 Increased expression of ICAM-1 in luminal endothelium of the aortas of ApoE^−/−^ mice. Representative images shown co-expression (yellow) of ICAM-1 (green) and CD31 (red) in luminal endothelium at areas without plaques A and at plaques B within the aortas of ApoE^−/−^ mice fed with high-fat diet (HFD+) compared with standard diet (HFD−). Images of individual staining with CD31 (red), ICAM-1 (green), and DAPI (nucleus) at plaque are also shown in B. Sizes of scale bars are shown.
