# Supplementary figures and images for: Directing cell delivery to murine atherosclerotic aortic lesions via targeting inflamed circulatory interface using nanocarriers
Source: Front Cardiovasc Med. 2025 Jun 24;12:1517320. doi: 10.3389/fcvm.2025.1517320 (PMC12234573; doi:10.3389/fcvm.2025.1517320)

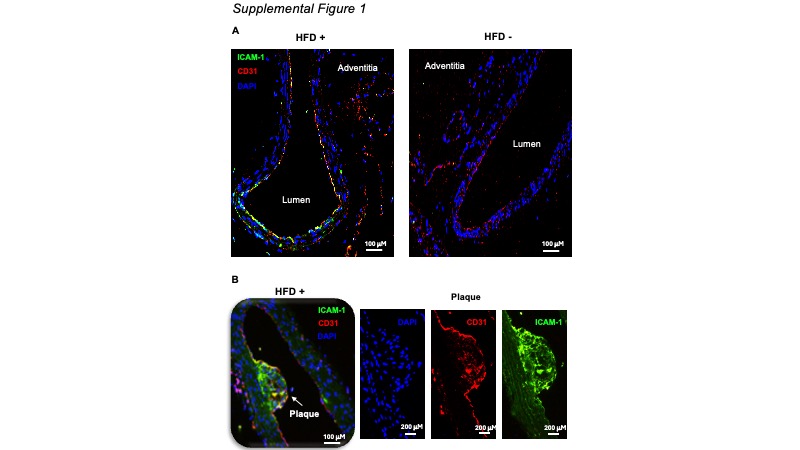

Supplement: Supplementary Figure 1 — Increased expression of ICAM-1 in luminal endothelium of the aortas of ApoE−/− mice. Representative images shown co-expression (yellow) of ICAM-1 (green) and CD31 (red) in luminal endothelium at areas without plaques A and at plaques B within the aortas of ApoE−/− mice fed with high-fat diet (HFD+) compared with standard diet (HFD−). Images of individual staining with CD31 (red), ICAM-1 (green), and DAPI (nucleus) at plaque are also shown in B. Sizes of scale bars are shown. [file Image1.jpeg]
